# Atypical cat-scratch disease with acute high-grade fever and neuropsychiatric symptoms: a case report

**DOI:** 10.3389/fvets.2026.1810698

**Published:** 2026-07-28

**Authors:** Xinhua Song, Xin Liu, Minjie Lou, Jinmei Xu, Xue Gong, Qing Yang, Guohua Chen, Junhua Mei

**Affiliations:** 1 Department of Neurology, Wuhan No. 1 Hospital, Wuhan, China; 2 Hubei University of Chinese Medicine, Wuhan, China; 3 Key Laboratory of Hubei Province for Neurological Disorders, Wuhan, China

**Keywords:** *Bartonella henselae*, case report, cat-scratch disease (CSD), metagenomic next-generation sequencing (mNGS), neurobartonellosis

## Abstract

**Objective:**

To report an atypical case of neurological cat-scratch disease (NCSD) presenting with acute-onset fever and prominent neuropsychiatric manifestations in an older adult.

**Patient:**

An 85-year-old East Asian man with a history of hypertension and coronary artery disease.

**Results:**

Three months after a cat scratch, the patient developed abrupt high-grade fever, followed by nocturnal delirium with visual hallucinations and a witnessed seizure-like episode, and later complained of occipital headache. Physical examination revealed mild bilateral axillary and left supraclavicular lymphadenopathy without focal neurologic deficits. Cerebrospinal fluid cultures and stains were negative for bacteria and fungi. Serum metagenomic next-generation sequencing (mNGS) detected *Bartonella henselae*. After antibiotic therapy, the fever and headache resolved.

**Conclusion:**

Cat-scratch disease should be considered as a rare but important infectious etiology in patients presenting with febrile illness accompanied by delirium or other neuropsychiatric symptoms. Clinicians should carefully inquire about recent cat exposure or scratch history and consider early pathogen-directed empiric antibiotic therapy to minimize diagnostic delay and improve outcomes.

## 1 Introduction

Cat-scratch disease (CSD) is a worldwide zoonotic bacterial infection caused by *Bartonella henselae*. Cats serve as the major reservoir host and are primarily infected by Ctenocephalides felis cat fleas. In addition to direct contact with cats, CSD can also be transmitted through flea bites and other vectors, especially in areas where flea infestations are common (1). The reported incidence is approximately 7.0 per 100,000 population (2). In addition to *Bartonella henselae*, other Bartonella species, such as *Bartonella bacilliformis* and *Bartonella quintana*, have also been implicated in Bartonella infections.

Clinically, 80–95% of patients with CSD present with fever. An erythematous papule typically develops at the site of a cat scratch within 3–5 days after inoculation, followed by regional lymphadenopathy 1–3 weeks later (1, 3). Approximately 20% of cases manifest atypical or disseminated disease, which may involve the eyes, nervous system, heart, liver, spleen, skin, or musculoskeletal system, and can lead to severe complications (4, 5). Neurological CSD (NCSD) accounts for an estimated 1–7% of cases. A wide spectrum of neurological manifestations has been described, including neuralgic amyotrophy (6), chronic inflammatory demyelinating polyneuropathy (7), Guillain Barré syndrome (8, 9), cranial nerve paralysis (10–12), peripheral vasculitic polyneuropathy (13), acute transverse myelopathy (14), neuroretinitis (15), encephalitis or encephalopathy (16), cerebral vasculitis or aneurysm (17, 18) and neuropsychiatric conditions (19–21).

Here, we report a patient who presented with acute-onset fever and prominent psychiatric symptoms, followed by headache. *Bartonella henselae* infection was confirmed by serum metagenomic next-generation sequencing (mNGS), providing clinical insight into the recognition of atypical CSD with predominant neuropsychiatric manifestations.

## 2 Case

An 85-year-old East Asian man presented with abrupt-onset high fever accompanied by altered mental status. Two days prior to admission, he developed a sudden high-grade fever with a maximum temperature of 40 °C, along with nocturnal incoherent speech. He also reported nausea, vomiting, anorexia, and diffuse body aches. He was initially evaluated in the neurology department of a local hospital, where no focal neurological deficits were identified. Emergency computed tomography (CT) scans of the head, chest, and abdomen were unremarkable, with no evidence of a focal infection or mass lesion.

At the night of hospitalization at the outside facility, he developed visual hallucinations, describing “smoke coming from the bed,” without accompanying auditory hallucinations or delusional thoughts. On the following day, a suspected seizure-like episode was witnessed by family members. The patient was found collapsed on the bathroom floor with his eyes open but unresponsive to verbal stimuli, without witnessed limb convulsions. No urinary or fecal incontinence was reported. After several minutes, he regained full consciousness, was able to communicate normally, and could stand and walk independently. He was amnestic for the event, which lasted approximately few minutes and resolved spontaneously.

Laboratory testing at the outside hospital revealed a neutrophil proportion of 72.5% (normal range, 40–75%), C-reactive protein of 96.79 mg/L (normal range, 0–10 mg/L), and procalcitonin of 0.618 ng/mL (normal range, 0.05–0.5 ng/mL). Despite empiric antimicrobial therapy at the local hospital, the specific antimicrobial agents were not documented in the available medical records. Recurrent fever persisted, and on the 5th day in hospital, he developed a left occipital headache. He denied recent upper respiratory symptoms, abdominal pain, chest pain, substance misuse, or head trauma. His medical history was significant for hypertension, for which he was taking amlodipine besylate 2.5 mg once daily, and coronary artery disease diagnosed in 2014 without ongoing pharmacologic treatment.

He was subsequently transferred and admitted to the Department of Neurology at our hospital. On admission, his temperature was 37.5 °C, pulse was 79 beats/min, and blood pressure was 155/80 mmHg. He was alert and oriented. Bedside cognitive testing revealed deficits in attention, comprehension, calculation, and short-term memory. The neck was supple. Motor strength was 5/5 throughout with normal tone. No pathological reflexes were elicited. Deep tendon reflexes were brisk but symmetric. Coordination testing could not be adequately assessed because of poor cooperation. There were no meningeal signs. Chest examination revealed coarse breath sounds bilaterally without crackles. There was tenderness over the left side of the neck. However, no palpable superficial lymphadenopathy was detected.

Initial laboratory investigations are summarized in Table 1. Autoimmune serologies and antineutrophil cytoplasmic antibodies were negative, and the remainder of the blood chemistry panel and urinalysis were within normal limits. On cognitive screening, the Mini-Mental State Examination (MMSE) score was 10/30 and the Montreal Cognitive Assessment (MoCA) score was 4/30.

**Table 1:** Initial laboratory findings.

Parameter	Result	Reference range
Hemoglobin	11.2 g/dL	13.0–17.5 g/dL
White blood cell count	6.92 × 10^9^/L	3.5–9.5 × 10^9^/L
Neutrophils	73%	40–75%
Lymphocytes	13.9%	20–50%
Monocytes	11.8%	3–10%
Eosinophils	1.2%	0.4–8.0%
Serum amyloid A	193.17 mg/L	0–10 mg/L
High-sensitivity C-reactive protein	69.29 mg/L	0–1 mg/L
Tumor necrosis factor-*α*	18.70 pg/mL	0–8.1 pg./mL
Interleukin-6	34.06 pg/mL	0–7 pg./mL
B-type natriuretic peptide	484.9 ng/L	0–100 ng/L
Serum albumin	27.5 g/L	40–55 g/L
D-dimer	1.76 mg/L	0–1 mg/L

**Figure 1:**
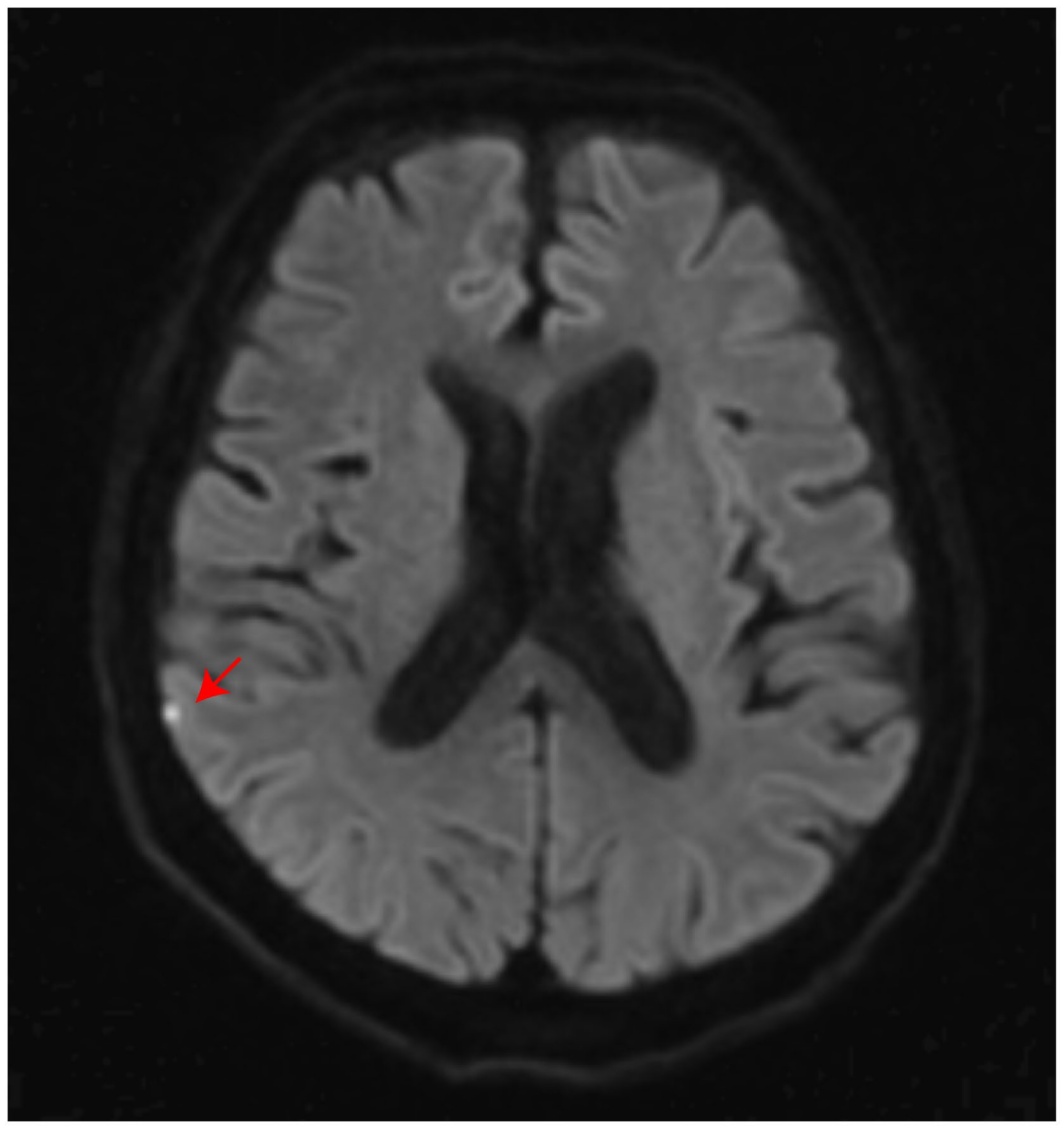
Brain magnetic resonance imaging findings. Diffusion-weighted imaging showed a focal hyperintense lesion in the occipital lobe (red arrow).

**Figure 2:**
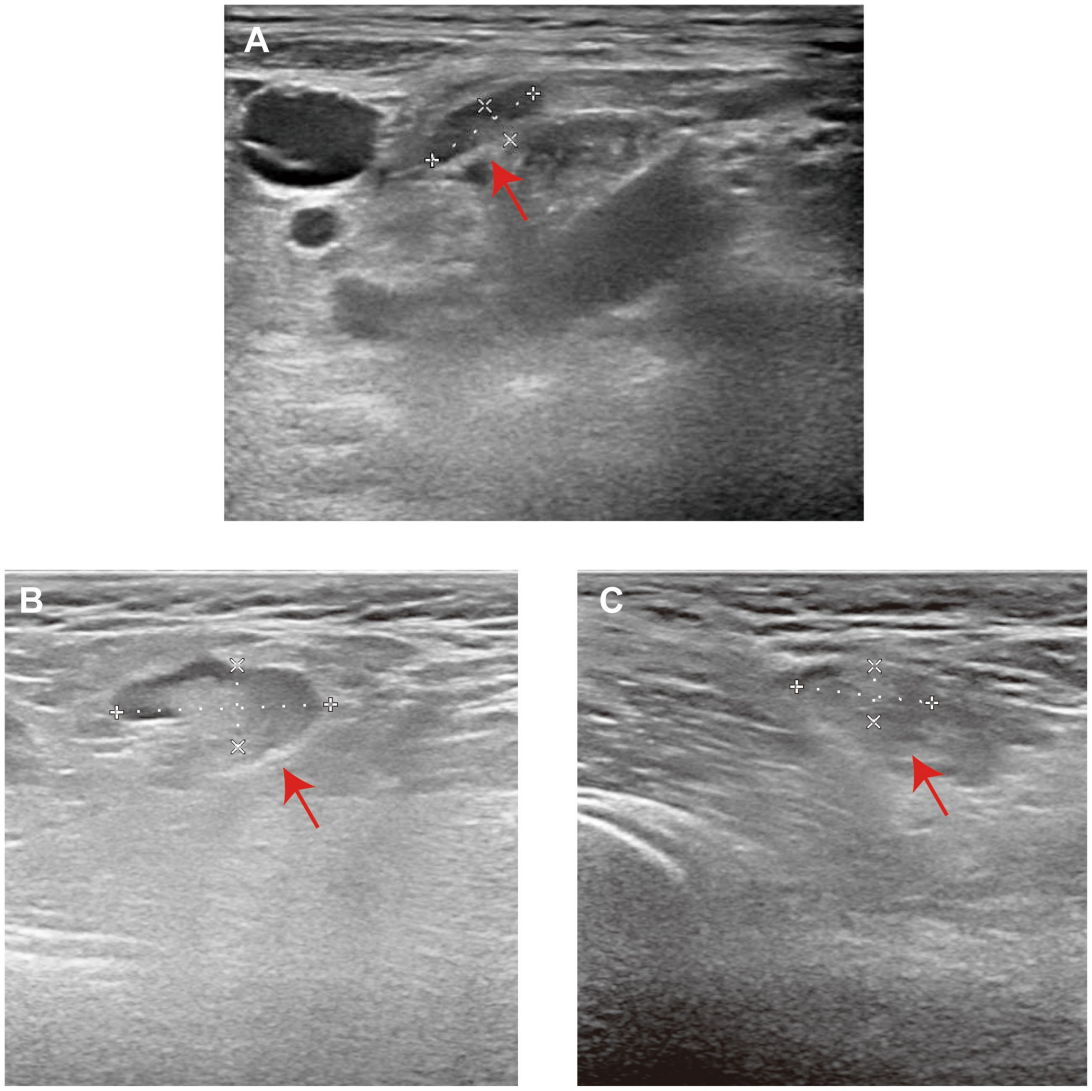
**(A)** Lymph nodes in the left supraclavicular fossa under Color Doppler ultrasound. **(B)** Lymph nodes in the left axilla under Color Doppler ultrasound. **(C)** Lymph nodes in the right axilla under Color Doppler ultrasound.

Chest CT demonstrated scattered inflammatory changes in both lungs with bilateral pleural effusions. Brain diffusion-weighted imaging (DWI) revealed a punctate hyperintense lesion in the right occipital lobe (Figure 1). Superficial lymph node ultrasound showed left supraclavicular fossa lymphadenectasis, left axillary lymphadenectasis with structural abnormality, and right axillary lymphadenectasis with no structural abnormality. Lymph nodes in the bilateral cervical and bilateral inguinal were found without any structural abnormality (Figure 2).

On the second day of hospitalization at our hospital, a lumbar puncture was obtained. Cerebrospinal fluid (CSF) analysis showed a leukocyte count of 1 × 10^⁶^/L, chloride level of 122 mmol/L (normal range, 120–130 mmol/L), glucose level of 2.9 mmol/L (normal range, 2.8–4.5 mmol/L), and protein level of 571 mg/L (normal range, 120–600 mg/L). CSF lactate dehydrogenase and adenosine deaminase were within normal ranges. Gram stain, fungal stain, and acid-fast staining were negative. CSF cryptococcal antigen testing was also negative. CSF cultures under both aerobic and anaerobic conditions were negative. CSF lactate dehydrogenase and adenosine deaminase were within normal ranges.

Initial differential diagnoses included transient ischemic attack (TIA), viral encephalitis, autoimmune encephalitis, and other common central nervous system infections. However, after detailed evaluation, based on the patient’s presentation of no clear focal neurological deficits, the possibility of TIA was excluded. As a result, intravenous meropenem therapy was initiated at a dose of 0.5 g every 8 h on hospital days 1–3. However, intermittent low-grade fever persisted. The patient resides in an urban area, where no relevant epidemics have been reported.

Metagenomic next-generation sequencing for pathogen detection was subsequently performed and returned positive for *Bartonella henselae*. We used blood as the sample type, and the sequencing method was metagenomic capture and profiling (MetaCAP), a probe-capture high-throughput sequencing technique. In our patient, *Bartonella henselae* was detected with a relative abundance of 6.53% and a detection confidence level of 99%. The detailed methods and results are provided in Supplementary material. With further review of his social history, his family reported that he kept a cat at home and frequently shared bowls and cups with the cat. He sustained a cat scratch with skin breakage and bleeding on the dorsum of his left hand 3 months earlier. On day 2 after exposure, the patient received one dose of human rabies immunoglobulin, 1,400 IU (BOHUI, China), and one dose of human rabies vaccine, Vero cell-based, approval number S20043090 (CHENGDA BIOTECHNOLOGY, China). A second dose of human rabies vaccine was administered on day 4 after exposure, with a 2-day interval between the first two vaccine doses. However, he did not complete the remaining three doses of the vaccination series because of fever. He was treated with moxifloxacin for 3 days, after which the fever did not recur.

Further epidemiological investigation revealed that the cat was a stray cat adopted by the patient. According to the local veterinarian’s assessment, the cat was approximately 1 month old at the time of the scratch, female, unspayed, and had outdoor access. The cat had not received preventive vaccination before the scratch but subsequently received two doses of vaccine. Fleas were observed on the cat when it was adopted, and deworming/ectoparasite control was performed; however, complete elimination of fleas could not be confirmed. The patient had no other pets, no other known animal contact, no other known flea or vector insect exposure, and no history of flea infestation in the residence. Based on this epidemiological history, the stray cat was considered the most likely source of infection.

**Figure 3:**
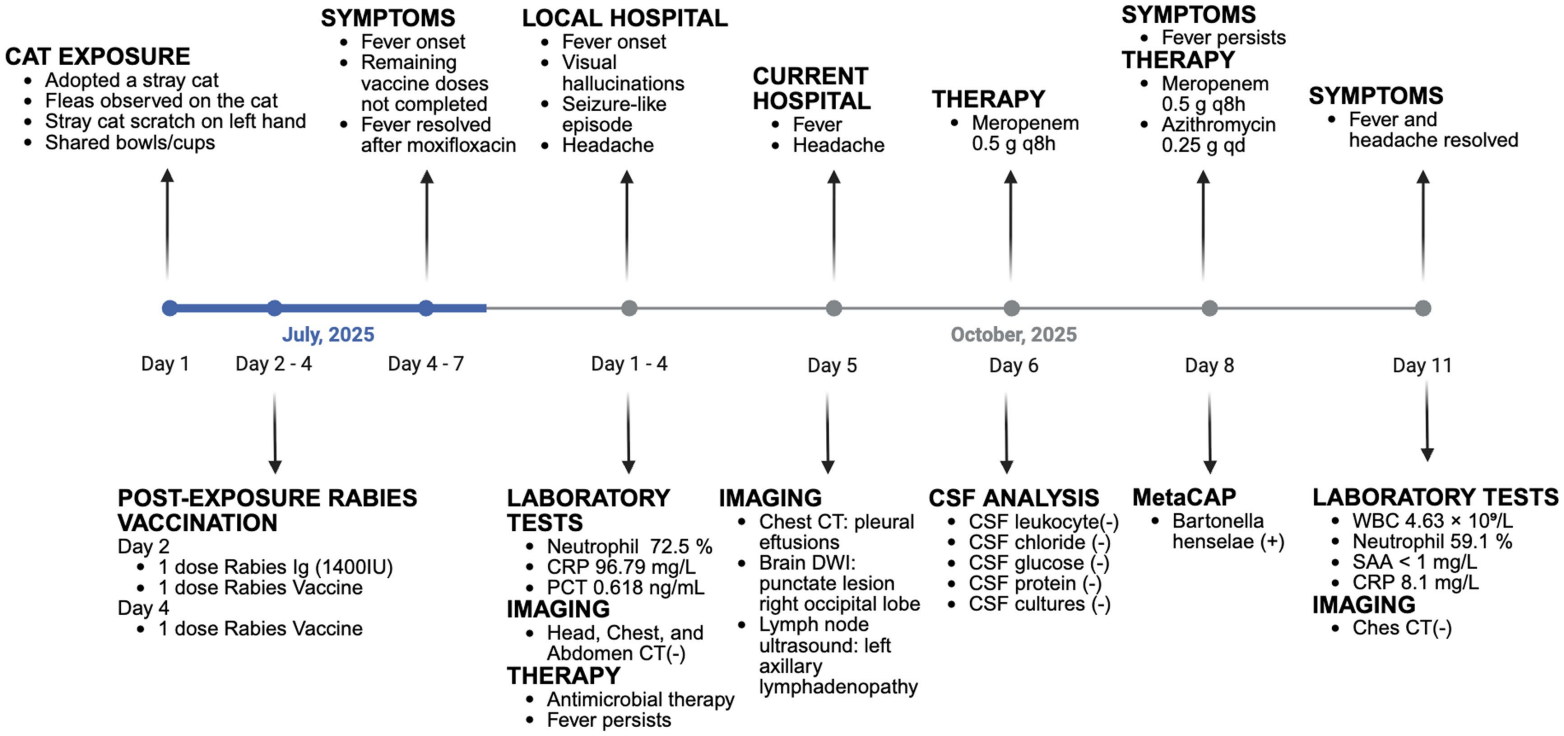
Timeline of clinical events, diagnostic findings, and treatment course. The timeline summarizes the patient’s cat exposure, post-exposure rabies prophylaxis, symptom progression, laboratory and imaging findings, antimicrobial therapy, metagenomic testing results, and clinical outcome.

Based on the microbiological findings, cat scratch disease was diagnosed. The antibiotic regimen was adjusted to intravenous meropenem 0.5 g every 12 h plus intravenous azithromycin 0.25 g once daily for 4 days on hospital days 4–7. Thereafter, fever and headache resolved, accompanied by a decline in inflammatory markers. Follow-up laboratory tests showed a WBC count of 4.63 × 10^⁹^/L (59.1% neutrophils, 26.9% lymphocytes, 8.0% monocytes, and 5.2% eosinophils), serum amyloid A < 1 mg/L (normal range, 0–10 mg/L), and C-reactive protein 8.1 mg/L (normal range, 0–10 mg/L). After discharge, the patient did not experience any further episodes of fever, nor did he develop any recurrent headaches or neuropsychiatric symptoms (Figure 3).

## 3 Discussion

Here, we report an atypical case of CSD with central nervous system involvement, presenting with acute-onset fever and prominent neuropsychiatric symptoms. The differential diagnosis of acute fever accompanied by altered mental status is broad and should include viral encephalitis, autoimmune encephalitis, bacterial or tuberculous central nervous system infection, metabolic- or drug-related delirium, and acute cerebrovascular events. However, CSD is an uncommon etiology and may be easily overlooked.

Given the patient’s presentation at the referral center, he is an elderly male with an acute onset, primarily presenting with fever and neuropsychiatric abnormalities. According to the Midnights principle, infection and stroke are the primary differential diagnoses. Although DWI showed a punctate hyperintense lesion in the right occipital lobe, TIA typically does not present with persistent imaging abnormalities. The symptoms of TIA are usually transient, resolving within minutes to hours, and imaging often does not show lasting lesions. Additionally, the patient did not present with typical focal neurological deficits, such as hemiparesis or aphasia, after onset. Considering the patient’s elevated inflammatory markers, the possibility of TIA was further excluded, and an infectious etiology seemed more likely. The DWI hyperintense lesion may be related to Bartonella infection.

CSD is more common in children and adolescents, with the highest incidence reported among those aged 7–12 years (2). CSD is rarely reported in elderly. We report a rare case of NCSD occurring in an elderly patient. Previous reports indicate that Bartonella infections are more common in immunocompromised hosts. Although the patient did not present with overt signs of immunosuppression, his advanced age suggests potential immune senescence. This could have been a contributing factor to the atypical neuropsychiatric manifestations of Bartonella infection in this case.

In NCSD, most patients develop fever approximately 1–2 weeks before the onset of neurological symptoms. Common neurological manifestations include headache and seizures, and severe cases may progress to status epilepticus (22, 23).

In contrast, NCSD presenting with prominent psychiatric symptoms as the initial or predominant manifestation has been reported only infrequently. Samarkos et al. (19) reported a 53-year-old man who developed confusion, expressive aphasia, and cognitive decline after a cat bite, accompanied by fever and left inguinal lymphadenopathy. Breitschwerdt et al. (20) described a 14-year-old boy who suddenly developed hallucinations and delusions, along with suicidal and homicidal ideation. Schaller et al. (21) reported a 41-year-old man who, following a tick bite, presented with an acute onset of mania, insomnia, and personality changes, accompanied by fever and painful axillary lymphadenopathy.

In our case, the patient developed nocturnal delirium and visual hallucinations at disease onset, and a subsequent seizure-like episode was witnessed by family members. Because electroencephalography was not performed during the clinical course, we could not definitively classify this event as an epileptic seizure. Given the early presentation with delirium, visual hallucinations, and headache, our case is consistent with an atypical neuropsychiatric phenotype within the NCSD spectrum.

While NCSD with neuropsychiatric symptoms is often considered rare, it may be more common than recognized, especially if clinicians include it in their differential diagnoses. This under recognition may contribute to its perceived rarity. This case emphasizes the need to consider NCSD in febrile patients with neuropsychiatric symptoms, particularly those with a history of cat exposure or scratch, to improve diagnostic accuracy and outcomes through earlier pathogen-directed therapy.

Reports suggest that approximately 90% of patients with CSD have a history of cat exposure, and about 60% report having been scratched or bitten by a cat (24). In our case, the patient did not volunteer a history of a cat scratch or bite at the initial visit. In fact, many patients provide such specific exposure details only when directly questioned. As noted above, typical CSD presents with fever and subacute regional lymphadenopathy. Case reports have also described varying degrees of lymphadenopathy in some NCSD patients (15), and fever and lymphadenopathy may precede neurological symptoms (25).

In our case, the patient complained of left-sided neck pain. However, the initial admission examination did not detect any palpable lymphadenopathy. Subsequent ultrasonography of superficial lymph nodes demonstrated enlargement of the left supraclavicular lymph node and bilateral axillary lymph nodes. Therefore, lymphadenopathy represents an important clinical clue when evaluating suspected atypical NCSD.

Regarding etiological diagnosis of *Bartonella henselae* infection, isolation of the organism from blood is challenging because of its fastidious growth requirements and low culture yield (26, 27). Serologic testing is commonly used as the preferred initial diagnostic approach. The indirect fluorescent antibody (IFA) assay has relatively high specificity but suboptimal sensitivity (28). A Japanese study reported that combining IFA with real-time PCR improves the positive detection rate of *Bartonella henselae* (29).

## 4 Conclusion

NCSD caused by *Bartonella henselae* is a rare but important etiology of febrile illness with neuropsychiatric manifestations, and timely recognition is associated with favorable outcomes. In patients presenting with fever accompanied by neurobehavioral disturbances, seizure-like episodes, or headache, the coexistence of regional lymphadenopathy should raise strong suspicion for NCSD. If further history-taking reveals exposure of the cat scratch or bite event, *Bartonella henselae* infection should be included in the differential diagnosis, and pathogen-directed empiric antibiotic therapy should be considered early after common causes have been excluded, even before definitive microbiological or serologic confirmation, to minimize diagnostic delay and improve prognosis.

## Data Availability

The raw sequencing data generated in this study have been deposited in the NCBI Sequence Read Archive (SRA) under BioProject accession number PRJNA1481182.
