# Supplementary material for: Atypical cat-scratch disease with acute high-grade fever and neuropsychiatric symptoms: a case report
Source: Front Vet Sci. 2026 Jul 28;13:1810698. doi: 10.3389/fvets.2026.1810698 (PMC13456991; doi:10.3389/fvets.2026.1810698)
Supplement: Supplementary file 1 [file Table_1.docx]

**Supplementary Material: Detailed Results of Metagenomic Next-Generation Sequencing (mNGS)**

Sample Type: Blood

Sequencing Method: Metagenomic capture and profiling (MetaCAP) is a widely used diagnostic tool in public laboratories, particularly for complex infections where traditional diagnostic methods may fail. This high-throughput sequencing method enables the detection of a wide range of pathogens from clinical samples, including bacteria, viruses, fungi, and parasites. The technology utilizes high-density probes to capture microbial nucleic acids and enrich them for sequencing. This method covers 21388 microbial species, including 11958 bacterial species, 7373 viral species, 1714 fungal species, and 343 parasitic species. The testing was performed at the Wuhan Center for Disease Control and Prevention, Public Health Service Center, a recognized public health laboratory.

The process begins with the collection of blood samples, which are then stored at low temperatures until DNA/RNA extraction. The microbial nucleic acids are extracted using standard kits (QIAGEN), ensuring high-quality DNA/RNA for downstream analysis. Following extraction, the MetaCAP method employs a probe-capture high-throughput sequencing technique. Specific probes are designed to bind to known microbial genomic sequences from bacteria, viruses, fungi, and parasites. These probes are hybridized with the microbial DNA/RNA in the sample, followed by magnetic bead-based capture to isolate the target sequences, enriching them for sequencing. The enriched DNA/RNA is amplified through PCR to ensure sufficient quantity for high-throughput sequencing on Illumina NovaSeq 6000. Sequencing generates millions of short DNA reads, which are then mapped against public microbial databases to identify the pathogens present. The relative abundance and detection confidence of each pathogen are calculated, and stringent quality control measures, including monitoring Q30 ratio (over 91%) and capture efficiency (91.76%), are applied to ensure the reliability of the results.

Detected Pathogens: The following pathogens were detected using metagenomic next-generation sequencing:

1.Bartonella henselae: Relative Abundance: 6.53%. Detection Confidence Level: 99%.

2.Epstein-Barr Virus (EBV): Relative Abundance: 93.67%; Detection Confidence Level: 99%.

3.Human Polyomavirus 2 (JC Polyomavirus): Relative Abundance: 2.11%; Detection Confidence Level: 86%.
